# Examining Adolescence as a Sensitive Period for High-Fat, High-Sugar Diet Exposure: A Systematic Review of the Animal Literature

**DOI:** 10.3389/fnins.2019.01108

**Published:** 2019-10-25

**Authors:** Susan Murray, Eunice Y. Chen

**Affiliations:** Department of Psychology, Temple University, Philadelphia, PA, United States

**Keywords:** fat, sugar, adolescence, memory, hippocampus

## Abstract

Animal studies suggest that poor nutrition (e.g., high-fat, high-sugar diets) may lead to impairments in cognitive functioning. Accumulating evidence suggests that the deleterious effects of these diets appear more pronounced in animals maintained on this diet early in life, consistent with the notion that the developing brain may be especially vulnerable to environmental insults. The current paper provides the first systematic review of studies comparing the effects of high-fat, high-sugar diet exposure during adolescence and adulthood on memory performance. The majority of studies (7/8) identified here report diet-induced memory problems when diet exposure began in adolescence but not adulthood. These findings lend support to the hypothesis that adolescence is a sensitive period during which palatable diets may contribute to negative neurocognitive effects. The current review explores putative mechanisms involved in diet-induced cognitive dysfunction and highlights promising areas for further research.

## Introduction

Dessert, pizza, and soda are the three primary sources of daily calorie intake for children and adolescents in the United States (Reedy and Krebs-Smith, 2010). Though the negative health risks associated with poor diet (e.g., diabetes and cardiovascular disease) are widely known, there is considerably less public awareness regarding the link between diet quality and cognitive functioning. Evidence from animal and human studies suggest that diets high in saturated fats and refined sugars are associated with impaired memory performance and hippocampal dysfunction (Greenwood and Winocur, 1990; Kanoski et al., 2007; Francis and Stevenson, 2011; Baym et al., 2014). Emerging evidence from the animal literature suggests that the negative effects of these diets may be more pronounced when exposure occurs during the adolescence, consistent with the notion that the developing brain is especially vulnerable to environmental insults (Schneider, 2013). Recent reviews (Yeomans, 2017; Davidson et al., 2019) have synthesized the literature regarding the effects of palatable diets on cognition, including reviews with a focus on the effects of diet exposure during adolescent development in particular (Reichelt, 2016; Reichelt and Rank, 2017; Del Olmo and Ruiz-Gayo, 2018). The current paper seeks to extend this literature by conducting the first systematic review of animal studies including groups exposed to high-fat, high-sugar diet during adolescence and adulthood and tested for memory.

## Methods

**Figure 1:**
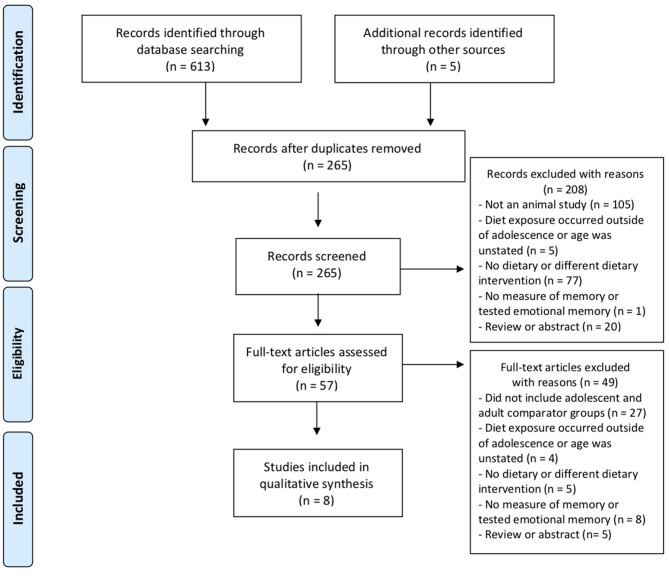
PRISMA flow diagram illustrating the process by which relevant studies were identified.

Relevant studies up to 5/12/2019 were identified through PubMed and Web of Science database searches using the terms “juvenile,” “adolescent,” “adolescence,” “diet,” “fat,” “sugar,” and “memory.” These search terms yielded a total of 613 results and an additional 5 articles were identified from reference lists (Figure 1). After removing duplicates (*n* = 353), 265 abstracts were screened and 208/265 excluded. Papers were excluded on the basis of the type of publication (i.e., reviews or abstracts were not included), if the study was conducted in humans, and aspects of study design. For example, diet exposure occurred outside of adolescence (approximately postnatal day 28–60 Spear, 2000), there was no diet manipulation or a different type of diet (e.g., protein restriction), or there was no memory task administered or the study assessed emotional memory which is thought to rely on the amygdala. This left a total of 57 full-text articles assessed for eligibility. Of these, 49 studies were excluded, leaving 8 eligible studies. Eligible studies included both adolescent and adult groups of rodents exposed to high-fat or high-sugar diets and tested for memory performance. Though calculating total N is complicated by the fact that some studies used separate cohorts of rodents to test different outcomes (e.g., to avoid the confound of exposure to behavioral testing on brain tissue), the eight studies included a minimum total of 309 rodents.

## Results

A total of eight studies including both adolescent and adult groups were identified (Table 1; Privitera et al., 2011; Boitard et al., 2012, 2014; Kendig et al., 2013; Valladolid-Acebes et al., 2013; Hsu et al., 2015; Klein et al., 2016; Labouesse et al., 2017). Six of these examined the effects of a high fat diet (HFD). Two assessed the effects of sucrose and one of these two also tested the effects of high fructose corn syrup (HFCS). Six of the eight studies included analysis of possible neural mechanisms. Adolescent diet exposure began between 3 and 6 weeks of age and adult diet exposure began between 8 and 12 weeks of age. Diet duration ranged significantly across studies, from 2 to 14 weeks. Only one of the eight studies included female subjects. Five of the eight studies included additional measures to eliminate potential confounds, such as locomotor activity, distance traveled, swimming speed, and time spent exploring. Of the eight studies identified, seven reported poorer performance across a range of cognitive tasks (Table 2) following diet exposure during adolescence but not adulthood (Privitera et al., 2011; Boitard et al., 2012, 2014; Valladolid-Acebes et al., 2013; Hsu et al., 2015; Klein et al., 2016; Labouesse et al., 2017). The one study that did not observe an age-dependent effect reported poorer cognitive performance following sucrose access regardless of age of exposure (Kendig et al., 2013). All animals were tested in adulthood including groups with diet exposure during adolescence.

**Table 1:** Description of the eight animal studies identified in the current review.

	**Species**	**Sex**	**Groups (at least 4 groups per study)**	**Diet duration**	**Age at testing**	**Behavioral measure**	**Behavioral findings**	**Neural findings**
Privitera et al. (2011)	Sprague Dawley rats	Male	Adol (3 wks) or Adult (8 wks) X HFD (60%) or Ctl (10%) *n* = 6 per group	2–3 wks	8 or 14 wks	CPP	CPP was not seen in adolescent HFD-fed groups when tested immediately or after several wks; CPP was seen in adolescent Ctl and adult HFD-fed groups	NA
Boitard et al. (2012)	C57BL/6J mice	Male	Adol (3 wks) or Adult (12 wks) X HFD (45%) or Ctl *n* = 27 Adol and 26 Adult	11 wks	14 or 23 wks	Radial maze	Mice fed a HFD in adolescence showed impaired performance on the recombination task but not the standard spatial discrimination task with no effect observed in adult rats	Adolescent mice fed a HFD showed reduced neurogenesis in the HP which was not seen in adult HFD-fed mice
Kendig et al. (2013)	Hooded Wistar rats	Male	Adol (4 wks) or Adult (9 wks) X Standard chow plus 10% sucrose solution 2 h daily or standard chow plus. 1% sodium saccharin 2 h daily *n* = 10 per group	4 wks	8 and 13 wks	MWM	Sucrose groups did not show different escape latencies during training; Sucrose groups showed impaired performance on probe trials; No effect of age	NA
Valladolid-Acebes et al. (2013)	C57BL/6J mice	Male	Adol (5 wks) or Adult (8 wks) X HFD (45%) or Ctl *n* = 12–15 per group	8 wks	13, 16, and 18 wks	NLR	Decreased discrimination ratios for NRL task in adolescent HFD groups even after calorie restriction; no effect in adults	Increased dendritic spine density in HP of adolescent HFD group but not adult HFD group
Boitard et al. (2014)	Wistar rats	Male	Adol (3 wks) or Adult (12 wks) X HFD (45%) or Ctl *n* = 10–14 per group	4, 8, or 12 wks	Varies	MWMR	No difference during learning or short term (2 h delay) trial of MWM; Adolescent HFD-fed animals showed poorer performance on the long-term (4 days) and reversal learning trials; No differences in adult groups	No evidence of increased inflammation following a HFD; after an immune challenge, adolescent HFD rats showed greater inflammation in the HP; not seen in adults
Hsu et al. (2015)	Sprague Dawley rats	Male	Adol (4 wks) or Adult (9 wks) X Chow plus *ad libitum* 11% sucrose or chow plus *ad libitum* 11% high-fructose corn syrup-55 or Ctl *n* = 12–13 per group	4 wks	8 or 13 wks	Barnes maze; Y maze	Adolescent HFCS group showed impaired acquisition and performance on the Barnes Maze Task whereas no effect was seen in adult groups; No effect of HFCS on Y maze performance in adolescent groups	Increased HP inflammation in adolescent HFCS group compared to age-matched controls with no differences seen between adult diet groups
Klein et al. (2016)	C57BL/6J mice	Female	Adol (6 wks) or Adult (10 wks) X HFD (60%) or Ctl *n* = 5–10 per group	12 or 14 wks	18 or 24 wks	MWMR	Adolescent sedentary rats fed a HFD during adolescence showed impaired flexible memory performance; Exercise prevented flexible memory impairments; HFD during adulthood did not lead to flexible memory impairments	Sedentary rats fed a HFD during adolescence showed decreased immature neurons in the HP; HFD was also associated with newborn neurons and survival of proliferating cells in the HP of adults
Labouesse et al. (2017)	C57BL/6J mice	Male	Adol (4 wks) or Adult (10 wks) X HFD (63%) or Ctl *n* = 9–10 per group	4 wks	10–17 or 15–21 wks	Y maze; MWM; T maze with reversal learning	Adolescent HFD group showed poorer performance on spatial working memory tasks (Y-maze and adapted MWM) and reversal learning in a T maze with no difference in learning T maze	Reduced in RELN cells in the mPFC of adolescent HFD group and impaired synaptic functioning; HFD adults showed reduced RELN in the HP

*Adol, adolescence; Wks, weeks; HFD, high fat diet; Ctl, control; CPP, conditioned place preference; NA, not applicable; HP, hippocampus; MWM, Morris Water Maze; NLR, novel location recognition; MWMR, reversal learning with MWM; RELN, Reelin; mPFC, medial prefrontal cortex*.

**Table 2:** Brief description of behavioral tests used to assess memory function in the studies identified via systematic review.

**Task**	**Brief description**
Morris Water Maze	Animals are trained to learn and remember visual cues to successfully locate a platform submerged in a pool of water
Barnes maze	Animals are trained to learn and remember visual cues to successfully locate a hidden escape under one of many holes surrounding a circular platform
Novel Location Recognition	Animals are presented with two objects, one of which has been moved from its previous location. If animals spend more time exploring the novel location, they are thought to have remembered that the other object has maintained its original location
Y-maze	Animals are allowed to explore the arms of a Y-shaped. If animals spend more time exploring one arm after another, they are thought to have remembered that they already explored the other^*†*^
Conditioned Place Preference	Animals are trained to associate a location with a rewarding or neutral stimuli and condition place preference is thought to be established when animals tend to prefer the location associated with reward in its absence
Radial Maze	Animals learn to associate one of eight arms in the radial-maze with a food reward
Water T-maze	Animals learn to associate one arm of the T-maze with escape
Reversal training^*^	New contingencies, such as a new (1) location for the hidden platform after training in the MWM or (2) arm of the radial-maze associated with a reward or (3) arm of the water T-maze associated with escape

† *The Y-maze used in Hsu et al. (2015) relied on animals learning to associate one arm of the maze with an escape, similar to the T-maze paradigm*.

* *Other tasks can be used to memory or cognitive flexibility*.

In a seminal study by Privitera et al. (2011), rats fed a HFD during adolescence failed to develop conditioned place preference compared to rats fed a control diet during adolescence and adult rats fed a HFD (Privitera et al., 2011). Conditioned place preference, mediated in part by the hippocampus (Hitchcock and Lattal, 2018), is assessed by exposing animals to two locations, one of which contains a reinforcer (in this case, Cheetos^®^) and measuring the time spent in the location previously associated with a reinforcer. Boitard et al. (2012) also reported impaired memory flexibility in animals fed a HFD in adolescence but not adulthood. Impaired memory flexibility, or the ability to update memory by learning novel contingencies after learning a different set of relationships, relies in part on hippocampus (Rossato et al., 2006). Indeed, this study reported a 23% reduction in hippocampal neurogenesis in mice fed a HFD in adolescence but not adulthood. In another study by Boitard et al. (2014), rats fed a HFD in adolescence also exhibited impaired memory flexibility as well as impaired long-term (4 days delay), but not short-term (2 h delay) spatial memory, suggesting possible deficits in memory consolidation. The results of this study also point to a possible role of neuroinflammation, as adolescent HFD animals exhibited elevated inflammatory cytokines within the hippocampus in response to an immune challenge. Both the behavioral and inflammation findings were not replicated in HFD-fed adult rats.

Given the role of the prefrontal cortex in cognitive flexibility (Kim et al., 2011) and the well-characterized maturation of this region during adolescence (Arain et al., 2013), research has also begun to explore the role of the prefrontal cortex in HFD-induced deficits. Labouesse et al. (2017) demonstrate impaired spatial working memory along with cognitive flexibility, assessed via reversal learning, in animals fed a HFD in adolescence. Briefly, reversal learning was assessed by training animals to associate either the right or left arm of a T-shaped maze in water with an escape platform. After acquisition, the location of the escape arm was switched. These behavioral findings were accompanied by a significant reduction in reelin, a regulator of synaptic plasticity, within the medial prefrontal cortex. Neither effect was observed when diet exposure was initiated adulthood, though adult HFD-exposed mice did exhibit a downregulation of reelin in the hippocampus (Labouesse et al., 2017). Given that the adult HFD group also showed significant increases in body weight compared to age-matched controls, these deficits appear to be driven by age of diet exposure and not obesity.

Impaired spatial memory, assessed using a novel location recognition paradigm with 1 and 24 h delays, has also been observed in adolescent mice fed a HFD, with no effects in an adult HFD group (Valladolid-Acebes et al., 2013). Moreover, though elevated leptin levels were observed in HFD-fed mice of both age groups, only HFD-fed adolescent mice showed evidence of leptin resistance. This is of particular relevance as leptin has been shown to influence synaptic plasticity within the hippocampus and improve memory (Harvey et al., 2006). Interestingly, this study also found increased dendritic spine density of hippocampal neurons in HFD-fed adolescent mice which was hypothesized to reflect a potential compensatory reorganization of neuronal architecture.

In an effort to determine whether physical activity might protect against or ameliorate diet-induced memory deficits, Klein et al. (2016) compared memory performance between HFD-fed adolescent animals with and without simultaneous access to a running wheel. This study corroborates earlier findings showing that HFD during adolescence is associated with poorer memory flexibility and fewer immature neurons in the hippocampus. Exercise was shown to prevent HFD-related impairments in memory flexibility and increase the survival of proliferating cells and newborn mature neurons in the hippocampus (Klein et al., 2016). Exercise introduced after HFD access did not improve memory flexibility in animals with HFD exposure in adulthood though adult mice did not show impairments in diet-related spatial learning or memory flexibility. This study did not include an adult comparator group with access to both exercise and a HFD, making it difficult to compare results directly. Of note, adult HFD exposure did affect the survival of proliferating cells and newborn neurons in the hippocampus; in the absence of a behavioral effect, the authors propose that this may indicate “an age-dependent utility of neurogenesis.” This echoes previously described findings of lower reelin concentrations in the hippocampus of adult HFD mice, as here too neuroadaptations were not associated with cognitive performance as it was assessed.

Findings regarding an age-dependent vulnerability for the neurocognitive effects of sugar appear limited and mixed. One study found that compared to age-matched rats fed a standard chow diet, both adolescent and young adult rats given 2 h of sucrose access daily showed poorer performance on the Morris Water Maze (MWM) (Kendig et al., 2013), indicating an effect of sucrose access but not age of exposure. The MWM is often used as a spatial memory task and entails that animals learn to use visual cues in their surrounding environment to locate a hidden platform in a pool of water. Notably, this study also reported impaired memory performance among sucrose-exposed animals after over 6 weeks of abstinence. In another study, exposure to a HFCS solution during adolescence was associated with impaired performance on the Barnes maze task, which is similar to the MWM in that animals must learn to use spatial cues to locate an escape box hidden beneath one of 18 holes surrounding a circular platform. Unlike the MWM, this task is not administered in water; instead, animals are motivated to find an escape with the addition of bright light and white noise. In addition to differences in memory performance, animals that consumed a HFCS in adolescence exhibited increased IL-1β and IL-6 relative to adolescent sucrose- and chow-fed controls, whereas no differences in memory or inflammation were observed between adult diet groups (Hsu et al., 2015). This study found no effect of adolescent HFCS exposure on performance on the Y maze task, which the authors argue suggests a hippocampal-specific effect of HFCS. Performance on the Y maze is sometimes assessed by recording the number of alternations between the right and left arms of a Y-shaped maze which are taken as indications that the animal remembers which arm it last traveled down. However, in this study, animals were trained to associate one of two arms with an escape box (much like the T-maze mentioned earlier but without a reversal training component). In contrast to Kendig et al. (2013), Hsu et al. (2015) did not find sucrose to significantly affect memory performance in either adolescent or adult groups despite similar concentrations of sucrose, ages at diet initiation, and diet exposure periods. However, rodents were provided limited sugar access in the study by Kendig et al. (2013) and limited access models have been shown to promote greater sucrose intake (Eikelboom and Hewitt, 2016). These two studies also included different animal strains which may contribute to differences in memory performance (Jonasson, 2005). These experiments also operationalized memory using different tasks. Taken together, these findings suggest that consumption of HFCS, but not sucrose, may exert a more negative effect on memory and neuroinflammation during adolescence than adulthood.

## Discussion

The current findings suggest that adolescence represents a window of sensitivity to the deleterious neurocognitive effects of HFD and HFCS. This finding was observed rather consistently and despite between-study variability in diet composition, age at diet exposure, duration of diet exposure, age at testing, and the use of different measures of memory.

Proposed mechanisms for adolescent diet-induced cognitive deficits include reduced neurogenesis, altered synaptic plasticity, neuroinflammation, and dysfunction of appetite-regulating hormones, such as leptin. The current findings are consistent with multiple studies showing a reduction in neurogenesis following HFD access during adolescence (Boitard et al., 2012; Klein et al., 2016; Vinuesa et al., 2016, 2018). Studies also show reductions in hippocampal brain derived neurotropic factor (BDNF), a key regulator of neurogenesis, and increased markers of apoptosis (i.e., cell death) in adolescent HFD-fed mice (Wu et al., 2018). Such findings may help to explain neuroimaging data showing brain atrophy, including reduced hippocampal gray matter, among rats fed a HFD in adolescence or young adulthood (Kalyan-Masih et al., 2016; Rollins et al., 2019). Adolescent sucrose access has been associated with reductions in neurogenesis or neuroproliferation in some (Gueye et al., 2018) but not all studies (Ferreira et al., 2018; Xu and Reichelt, 2018). Sucrose exposure during this developmental period has also been shown to result in reductions in hippocampal parvalbumin-containing ⋎-aminobutyric acid (GABA)-ergic interneurons, thought to be critical for memory processes (Fuchs et al., 2007; Ognjanovski et al., 2017) and implicated in Alzheimer's disease pathology (Zallo et al., 2018).

Neuroinflammation has been proposed as another potential mediator of HFD-induced memory disturbance in adolescent animals though findings are mixed. As mentioned earlier, Boitard et al. (2014) report elevated hippocampal inflammation in response to an immune challenge in adolescent HFD-fed animals but no difference in basal markers of inflammation (Boitard et al., 2014). Mice fed a HFD in adolescence show evidence of microglial activation along with increased inflammation in the hippocampus (Vinuesa et al., 2018; Wu et al., 2018). In contrast, some studies have not observed significant differences in inflammatory biomarkers following adolescent HFD access (Kaczmarczyk et al., 2013; Wang et al., 2015). Recent evidence suggests that aged animals may be more susceptible to neuroinflammation following access to a cafeteria diet (Teixeira et al., 2019), suggesting an additional developmental period of vulnerability to high fat, high sugar diets.

Changes in synaptic plasticity, as reported by Labouesse et al. (2017), have also been documented previously following adolescent exposure to HFD. Hippocampal slices from adolescent mice fed a HFD for only 48 h showed partial inhibition of LTP, with particular impairment in LTP maintenance (Contreras et al., 2017). Moreover, adolescent HFD exposure has also been shown to result in synaptic remodeling and a 50% decrease in the Shank2 protein involved in spine morphogenesis (Vinuesa et al., 2018). Though the effects of a HFD on the hippocampus have been well-studied, Labouesse et al. (2017) also points to a role for the medial prefrontal cortex in diet-induced memory deficits. Given that these regions are known to form a functional system (Bizon et al., 2012), future research exploring how palatable diets affect cognitive development at the circuit level using a systems neuroscience approach may be insightful.

Many of the behavioral and neural outcomes described here have been reported in adult animals following access to palatable diets. For example, impaired reversal learning and reduced BDNF have been reported in adult animals fed a HFD (Kanoski et al., 2007) and signs of leptin resistance in diet-induced obesity have been observed independently of age (Sainz et al., 2015). Moreover, studies in the current review show evidence of neural changes in animals with HFD access beginning in adulthood. Therefore, it has been proposed that adult animals may have a higher threshold for sensitivity to diet-induced cognitive deficits such that higher fat content or longer diet duration may be necessary to observe behavioral differences (Boitard et al., 2012; Valladolid-Acebes et al., 2013).

Though Klein et al. (2016) suggest that exercise may help to prevent the deleterious effects of HFD exposure during adolescence, another promising intervention is switching animals to a lower fat diet (Kaczmarczyk et al., 2013; Boitard et al., 2016; Sims-Robinson et al., 2016). However, some data suggest long-term neurocognitive effects of adolescent exposure to palatable diets even after prolonged periods (e.g., up to 61 weeks) of diet abstinence (Kendig et al., 2013; Reichelt et al., 2015; Wang et al., 2015; Noble et al., 2019). Two of these studies examined the effects of sucrose (Kendig et al., 2013; Reichelt et al., 2015), one examined the effects of HFCS (Noble et al., 2019), and one examined the effects of a high fat diet (Wang et al., 2015). Additional research is needed to better understand potential long-term effects of adolescent consumption of fat and sugar on risk for cognitive decline in late life as well as to identify effective approaches prevention and intervention. One candidate for study may be working memory training as this has been shown to improve reference memory, reversal learning, and synaptic plasticity in rodents (Ioakeimidis et al., 2018).

The current set of findings are not without limitations. First, with a few exceptions (Boitard et al., 2012; Hsu et al., 2015), the studies described here did not directly compare outcomes between adolescent and adult groups and instead compared diet groups with their age-matched counterparts. In addition, age at testing varied between adolescent and adult groups; if diet duration is the constant for both groups and testing occurs directly following diet exposure, adolescent animals will be tested earlier in development relative to adults. However, typically, younger cohorts show superior memory performance relative to older cohorts (Lindner, 1997; Mizoguchi et al., 2010; Johnson and Wilbrecht, 2011), making it unlikely that this age discrepancy at testing would influence these findings. It is also worth noting that reversal learning tasks may reflect a number of cognitive functions, including inhibition, attention, information processing, and decision-making regarding search strategies, that may contribute to poorer task performance separate from or because of their contribution to memory processes. An additional limitation of the current research is the paucity of studies including female mice; future research examining sex differences in adolescent exposure to high-fat, high-sugar diets is warranted given evidence of sex differences in memory in rodent models (Jonasson, 2005). It should also be noted that some HFD formulations derive a sizeable proportion of the carbohydrate content from sucrose, which may confound findings. Moreover, fat source appears to differentially affect memory outcomes, with research showing that polyunsaturated fats improve memory and increase BDNF in adolescent animals (Dos Santos et al., 2018).

As pointed out by Hooijmans et al. (2014), systematic reviews of laboratory animal experiments are fairly uncommon and assessing risk of bias using the guidelines this group of authors set forth is somewhat challenging as certain information is often unstated in animal studies. For example, few studies included here stipulated that group assignment was based on random allocation and that assessments were conducted by investigators blind to experimental conditions. Future studies should include such information to assist in study evaluation. At the review-level, it is possible that the current search strategy did not capture all relevant studies, though two search databases were used. It is also quite possible that publication bias resulted in a skewed set of findings presented here, though one study included here did not observe an effect of age.

## Conclusions and Future Directions

Current dietary recommendations encourage limiting intake of added sugars and saturated fat throughout the lifespan. The findings reviewed here suggest that additional awareness could be raised about the intake of these diets during adolescence in particular. However, efforts to translate animal findings are needed to inform public policy initiatives. While available data are generally consistent with the hypothesis that diets high in fat and sugar may be associated with poorer neuropsychological performance in youth (Junger and van Kampen, 2010; Francis and Stevenson, 2011; Gibson et al., 2013; Baym et al., 2014), more studies are needed. Memory function is essential for supporting learning and has implications for academic achievement and everyday functioning; therefore, understanding the impact of high-fat, high-sugar diets on cognitive development throughout the lifespan remains an important area for further study.

## Author Contributions

SM conducted the systematic review of the literature. SM and EC contributed to the final version of the manuscript. EC supervised the project.

### Conflict of Interest

The authors declare that the research was conducted in the absence of any commercial or financial relationships that could be construed as a potential conflict of interest.
